# Age, period and cohort effects and the predictors of physical activity and sedentary behaviour among Chinese children, from 2004 to 2011

**DOI:** 10.1186/s12889-017-4215-x

**Published:** 2017-04-24

**Authors:** Xinping Wei, Yu Zang, Xiaodong Jia, Xiangui He, Shurong Zou, Hui Wang, Meihua Shen, Jiajie Zang

**Affiliations:** 1 Gu Mei Community Service Center, 668 Longming Road, Shanghai, 200010 People’s Republic of China; 2 Department of Neurology, the Affiliated Hospital of North China University of Science and Technology, No.73 south construction road, Tangshan, Hebei People’s Republic of China; 3 Department of Nutrition Hygiene, Division of Health Risk Factor Monitoring and Control, Shanghai Municipal Center for Disease Control and Prevention, 1380 West Zhongshan Road, Changning District, Shanghai, 200336 People’s Republic of China; 4 Department of Preventative Ophthalmology, Shanghai Eye Disease Prevention and Treatment Center, Shanghai Eye Hospital, Shanghai, 200040 People’s Republic of China; 5 Department of Epidemiology, School of Public Health, Nanjing Medical University, 101 Longmian Ave, Jiangning District, Nanjing, 211166 Jiangsu People’s Republic of China; 6 Department of Intensive Care Unit, Shanghai Provincial Crops Hospital, Chinese People’s Armed Police Forces, 831 Hongxu Road, Shanghai, 201103 People’s Republic of China; 7 Key Laboratory of Biomedical Information, Engineering, Ministry of Education, Institute of Mitochondrial Biology and Medicine, Xi’an Jiaotong University School of Life Science and Technology, Xi’an, 710049 People’s Republic of China

**Keywords:** Physical activity, Age, Period, Cohort, Children, China

## Abstract

**Background:**

Very few studies have explored the effects of age, time period, and cohort in association with biological, behavioral, economic, and environmental factors predictors on physical activity (PA) and sedentary behaviour (SB) among Chinese children.

**Methods:**

We used data from a cohort study of the China Health and Nutrition Survey (CHNS) between 2004 and 2011 (2004, 2006, 2009 and 2011). The outcomes of interest were metabolic equivalent of task (MET) hours per week from both active and sedentary activities. Age, gender, individual characteristics, household size, asset ownership, and urbanisation were included as covariates. Age, period and cohort effects analyses for PA and SB of children (6–17 y, *n* = 3528) was conducted to explicitly assess differences in PA and SB due to age vs. period effects, and implicitly assess differences by cohorts due to the period-specific experiences across individuals of varying ages.

**Results:**

The mean age of the sample in each time point fluctuated from 12.6 to 11.3 years and PA slightly decreased from 50.0 ± 63.2 MET hours per week (MET-hr./wk) in 2004 to 47.1 ± 54.9 MET-hr./wk. in 2011. However, SB increased from 31.8 ± 22.0 MET-hr./wk. to 37.6 ± 22.2 MET-hr./wk. Girls had lower PA and higher SB levels than boys. Controlling for age effects, marginal period effects on PA were observed in some survey years. Higher levels of urbanisation and number of household computers served as negative and positive predictors for PA and SB, respectively. Higher household income was a positive predictor of SB. Surprisingly, bigger household size was the only negative predictor of SB (*P* < 0.05).

**Conclusions:**

This longitudinal study followed a large cohort of children over a significant period of their childhood. We observed potential age and secular trends in PA levels. Higher community urbanisation and number of home computers were associated with both PA and SB levels. Larger household size was the only factor that was negatively associated with SB. These findings shed light on health policy and preventative health strategies for China and other countries that are now facing similar public health challenges.

**Electronic supplementary material:**

The online version of this article (doi:10.1186/s12889-017-4215-x) contains supplementary material, which is available to authorized users.

## Background

Many studies suggest that physical activity levels have decreased significantly in recent years. A total of only 35.6% students spent more than 1 h/day performing moderate to vigorous physical activity (MVPA) during school, and 34.9% spent more than1 h/day performing MVPA outside school hours in Beijing, China [1]. The inexorable rise in obesity has led to widespread calls for regular monitoring of child and adolescent weight in order to counter rising prevalence of obesity worldwide [2, 3]. Childhood obesity can precipitate adverse health and social consequences such as adult obesity, cardiovascular diseases, and type 2 diabetes mellitus [4]. Since physical activity (PA) contributes towards 18–29% of daily energy expenditure, it has become an important focus for research into targeting very high rates of childhood obesity [5].

It is widely accepted that engaging in regular physical activity is positively associated with a range of beneficial childhood health and fitness outcomes [6, 7]. Furthermore, it helps reduce the risk of non-communicable diseases (NCDs) in adults such as cardiovascular and cerebrovascular diseases, diabetes, obesity, hypertension, cognitive function and certain cancers [8, 9]. However, while the benefits of physical activity are well accepted, unprecedented social, economic, and educational system changes in China have been associated with increasingly lower levels of PA and higher levels of SB in Chinese children and adolescents. As economic and technological improvements have facilitated domestic housework and transportation, this has resulted in an increasingly sedentary lifestyle (e.g., watching television, playing video games, studying, and internet browsing) across the country in recent years [10].

There is public health concern that sedentary behaviours among children and adolescents may displace the time available for participation in PA, resulting in overall lower energy expenditure and consequent chronic health outcomes that are independent of PA [11–15]. Hence, it is important to evaluate both PA and SB separately.

Engagement in various types or forms of childhood PA is likely related to age, life-course, socioeconomic factors, asset ownership, the built environment, and degree of urbanisation. Therefore, measurement of these factors is important for increasing our understanding of how changes in PA may alter risk for obesity or disease. However, PA data for children and adolescents available in China are often cross-sectional [16–18]. Indeed, previous studies using such cross-sectional data to study epidemiological associations between exposures and PA between age groups may represent differences between birth cohort years rather than effects of exposures, which can be confounding [16–18]. Although there have been prospective studies of PA in China [19–21], these studies did not assess the period and birth cohort effects over and above age groups [19–21]. Thus, it is difficult to distinguish between the effects of aging, cohorts, and period when either a cross-sectional or longitudinal design is employed.

To address these gaps, this study describes changes in PA and SB among Chinese children and adolescents using longitudinal data covering a period of 8 years. We explicitly assessed the differences in PA within individuals over time (age effect) and population-wide differences in PA overtime (period effect), and implicitly assessed the differences in the experienced period effect across individuals of varying ages (cohort effect). We performed this analysis while adjusting for social, economic, and environmental factors over time to determine how these factors contribute to changes in PA levels in Chinese children.

## Methods

### Data

The China Health and Nutrition Survey (CHNS) is a representative, prospective household-based study that includes multiple ages and cohorts across nine rounds of surveys between 1989 and 2011 in nine diverse provinces (Guangxi, Guizhou, Heilongjiang, Henan, Hubei, Hunan, Jiangsu, Liaoning, and Shandong) and three large cities (Beijing, Shanghai, and Chongqing were added in 2011) [22]. A multistage, stratified sampling design was used to ensure that the CHNS provided representation of rural, urban, and suburban areas that varied substantially by geography, economic development, public resources, and health indicators [22].

We believe this is the only large-scale, longitudinal study of its kind in China. Details of the study design have been described elsewhere [23].

Information on travel and active leisure activities was not available until 1997, while information on sedentary leisure activities was not collected until 2004 (e.g., watching TV). As a result, we focused on childhood PA using four rounds of comparable survey data collected in 2004, 2006, 2009, and 2011. Additionally, observations were excluded from the analysis if a participant was currently disabled at the time of data collection. We focused our analyses on children, defined here as between 6 to 17 (less than 18) years old.

Our final analysis included 3528 participants, with an average of 1.5 observations collected per subject (5864 observations). We excluded participants with only one observation. The data are reflective of an unbalanced panel with some individuals being absent in certain years and returning in future years as the sample was replenished over time. However, relative to previous rounds of data collection, participant retention was between 80% and 88% across all surveys after 2004 [22, 23].

### Outcomes

The outcomes of interest were the metabolic equivalent of task (MET) hours per week (denoted as MET-hrs/wk). MET is defined as the ratio of a person’s working metabolic rate relative to his or her resting (basal) metabolic rate [24]. Therefore, the MET-hrs/week measurement accounts for both the average intensity of each activity (or subactivity) and the time spent in each activity.

Due to our interest in measuring the changes in PA and SB across time periods, the primary outcome used here was limited to active MET-hrs/wk. from domestic, travel, leisure, work, and in-school activities, while sedentary MET-hrs/wk. were calculated from watching TV, videos or movies, board games, online-surfing or gaming, reading, playing chess or with dolls, and studying. We defined this as “PA” and “SB” in the results. First, each reported activity was assigned a MET value using the Compendium of Energy Expenditures for Youth for children. Average MET values were used for activity categories such as ball sports or other sports. The MET value for each activity was then multiplied by the total time spent per week (hrs/wk) in the activity, resulting in the METhrs/ wk. measurement. Details on how these values were calculated are described elsewhere [25, 26].

### Covariates

#### Age and gender

Age in the baseline survey was used to define age groups of 6-8y, 9-11y, 12-14y, and 15-17y for analysis which represented children and adolescents from junior primary school, senior primary school, middle school, and high school. Gender was also considered as a main covariate associated with PA and SB.

#### Urbanisation

We calculated a multi-component urbanicity index from community survey data [27, 28], which reflected community infrastructure, population size and density, and economic and environmental characteristics. The scale illustrated misclassification using the traditional urban-rural dichotomy, and was able to detect differences in urbanicity, both between communities and across time. Furthermore, using a continuous measure of urbanicity allowed for better illustrations of the relationships between urbanicity and health. An increase in the value of the index over time represents greater urbanization.

#### Asset ownership

We were interested in the ownership of certain technologies or equipment related to PA and therefore included measurements of bicycle ownership, vehicle ownership, TV and computer ownership, and the number of computers at a household level.

#### Individual and family factors

We included individual level measures that may be associated with PA levels, which were weight status (normal weight vs. overweight/obese, which were defined using BMI cut-off points [29] for Chinese children and adolescents of 6–17 years old). We also accounted for household income (we generated ranked data by the quintiles in each wave) and household size (number of persons reported to be living in the household at the time of the survey) as potentially influential factors of PA.

### Statistical analysis

Due to potential differences related to gender, we reported the PA and SB outcomes (active MET-hrs/wk. and sedentary MET-hrs/wk) and explanatory variables (age, percentage of males, being overweight/obese) as mean ± SD or percentage stratified by gender for each survey year.

As the CHNS is a longitudinal study, attrition, migration, modifications to the sampling methods (including loss to follow-up and replenishment) over time, and other factors may result in cohort membership varying over time. In order to limit potential bias as a result of possible sample selection issues, we used a Heckman-two-step approach [30] to calculate the “inverse Mill’s ratio” for inclusion as a time-varying variable in all subsequent models described. The “inverse Mill’s ratio” is the inverse of the predicted probability that an individual was included in each survey given his/her province, community urbanicity, and their interaction.

A series of longitudinal mixed effects models with fixed and random individual-level effects and random slopes was used to explicitly assess differences in PA within individuals over time (age effect) and population-wide differences in PA overtime (period effect). Finally, we implicitly assessed the differences in the experienced period effect across individuals of varying ages (cohort effect). Mixed effects models were chosen because they can accommodate imbalanced data and continuous covariates, and because the hierarchical nature of the models overcomes the identifiability problem by not assuming that age-period-cohort (APC) effects are linear and additive at the same level of analysis [31, 32].

We employed four models. Model 1 considers fundamental factors of age, indicator variables representing survey year, age interaction with survey year and gender. The year coefficients describe the period effect and interaction terms describe how period effects may differ by age (cohort effect). Model 2 included the inverse Mill’s Ratio and community urbanicity index into model 1. We intended to estimate how these factors explain some of the period effects. Model 3 included individual and family level covariates (weight status and household size for example), asset ownership covariates (bicycle ownership and TV/computer ownership for example) into model 1. In addition to providing information about how these variables related to physical activity, comparing this model with the basic model provides information as to whether these individual factors can explain some of the period effects. Model 4 includes the full set of variables. All models were conducted using Stata’s XTMIXED program [33]. Results were considered significant if *P* < 0.05.

## Results

The total sample sizes of children in the study were 1692, 1357, 1198, and 1617 in 2004, 2006, 2009, and 2011 for each year. Cross-sectional analysis across survey years showed that the mean age of the included sample remained stable around from 12.5 ± 3.3 to 11.3 ± 3.3 years (Table 1). PA levels among Chinese children slightly decreased in MET hours across the 8-year period, from 50.0 ± 63.2 MET-hrs/wk. in 2004 to 47.1 ± 54.9 MET-hrs/wk. in 2011. The trends were similar for boys and girls. In contrast, SB among Chinese children increased in those years, from 31.8 ± 22.0 MET-hrs/wk. in 2004 to 37.6 ± 22.2 MET-hrs/wk. in 2011. The percentage of SB from total PA increased from 38.5% to 44.4%, from 35.8% to 41.6%, and from 40.1% to 45.7% among all boys and girls, for the different study years respectively. Meanwhile, the prevalence of being overweight and obese in Chinese children increased between 2004 and 2011 from 11.3% to 19.8%, from 11.7% to 23.4%, from 10.8% to 15.9% among all boys and girls, for the different study years respectively.

**Table 1:** Cross-sectional univariate descriptives of the China Health and Nutrition Survey across survey years, mean ± SD

	Survey years			
	2004	2006	2009	2011
All Participants(N)	1692	1357	1198	1617
Age	12.45 ± 3.33	11.91 ± 3.36	11.59 ± 3.19	11.33 ± 3.33
Male(%)	53.0	53.4	55.8	51.1
Overweight/Obese(%)	11.3	12.0	14.5	19.8
PA MET-hours/week	50.00 ± 63.15	47.56 ± 61.78	46.51 ± 56.73	47.14 ± 54.86
SB MET-hours/week	31.76 ± 22.00	34.85 ± 21.00	35.54 ± 21.83	37.62 ± 22.24
% SB in total PA	38.5	42.3	43.3	44.4
Male(N)	896	725	668	826
Age	12.57 ± 3.41	11.86 ± 3.40	11.57 ± 3.23	11.29 ± 3.31
Overweight/Obese(%)	11.7	13.3	16.4	23.4
PA MET-hours/week	57.15 ± 68.65	54.30 ± 68.07	52.66 ± 61.07	53.56 ± 60.52
SB MET-hours/week	31.89 ± 22.89	36.32 ± 23.05	35.06 ± 21.62	38.10 ± 24.48
% SB in total PA	35.8	40.1	40.0	41.6
Female(N)	796	632	530	791
Age	12.30 ± 3.22	11.97 ± 3.31	11.62 ± 3.15	11.38 ± 3.36
Overweight/Obese(%)^a^	10.8	10.5	12.0	15.9
PA MET-hours/week	47.24 ± 57.27	45.46 ± 54.98	43.66 ± 51.34	44.13 ± 48.49
SB MET-hours/week	31.61 ± 20.97	33.17 ± 18.24	36.16 ± 22.10	37.11 ± 19.62
% SB in total PA	40.1	42.2	45.3	45.7

^a^Using the criteria of overweight and obesity for Chinese children

*PA* physical activity, *SB* sedentary behaviour

### Physical activity (PA)

**Fig. 1:**
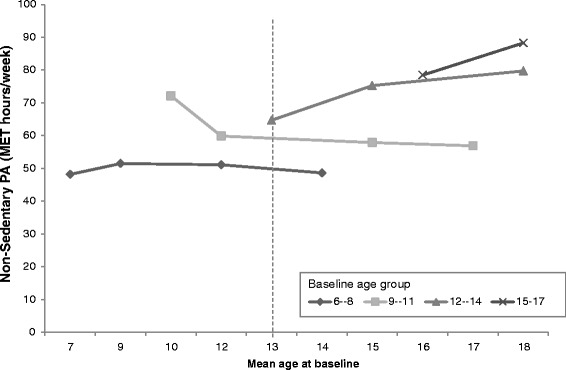
Age trends in mean physical activity among boys in the CHNS by baseline age groups (MET-hrs/wk). Notes: Points represent PA level of boys at the mean age for the age group in each survey year (2004, 2006, 2009, 2011). The vertical dotted line illustrates the difference in estimated PA level for a 13-year old boy in 2004 vs 13-year old boy in 2006 vs a 13-year old boy in 2009 (cohort effect).

**Fig. 2:**
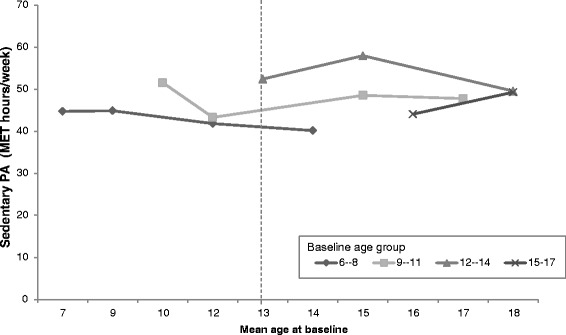
Age trends in mean physical activity among girls in the CHNS by baseline age groups (MET-hrs/wk). Notes: Points represent PA level of female children at the mean age for the age group in each survey year (2004, 2006, 2009, 2011). The vertical dotted line illustrates the difference in estimated PA level for a 13-year old girl in 2004 vs 13-year old girl in 2006 vs a 13-year old girl in 2009 (cohort effect)

In Fig. 1, PA levels in boys from the four baseline age groups are shown according to the mean age of the group by each survey year. Each point represents a survey year, and the lines span the 8-year period over which the PA data was collected. Younger boys had lower initial PA, and with age, PA levels changed differently across baseline age groups. The relationship between age and mean PA is non-linear. Meanwhile, the vertical dotted line illustrates the difference in estimated PA level for 13-year old boys in 2004 (65 MET-hr./wk) compared to 13-year old boys in 2006 (60 MET-hr./wk), and in 2011 (50 MET-hr./wk). This is deemed the cohort effect. PA levels of girls in the four baseline age groups are also shown according to the mean age of the group by each survey year in Fig. 2. A similar trend was observed for girls with respect to reduced PA levels while the cohort effect was also observed.

The non-linear age-related patterns of reductions in PA were confirmed by the longitudinal analysis. An age effect was not observed by adding age-square into the models. When adding age-square into the models, we found the coefficients for age and age-square contrasted with each other. Moreover, the age trend was not the same when different baseline age groups were examined (Fig. 1). Controlling for age, we found that being female was negatively associated with PA. The model results showed that period effects were positively associated with PA in 2011, when controlling for age and gender (Table 2, model 1). However, the period effect was extinguished after we adjusted for other covariates. The interaction between survey year and age (*p* < 0.05) was observed in 2011 (Table 2, model 1). In model 2, higher community urbanicity was found as a negative predictor of PA (Table 2, model 2). In model 3, we found that the addition of one extra computer per family resulted in a change in PA by −4.80 (−9.03,-0.57) MET-hr./wk. After adjusting for potential confounding effects of age, gender, the interaction of 2011 and age, and community urbanicity, the number of computers per family was identified as a negatively associated factor of PA (Table 2, model 4).

**Table 2:** Mixed effects estimates on physical activity (PA) levels (MET-hrs/wk) among Chinese children, coefficient (95% CI)

PA	Model 1	Model 2	Model 3	Model 4
Age	**6.12(5.28,6.96)**	**6.21(5.36,7.07)**	**5.6(4.72,6.49)**	**5.75(4.84,6.65)**
Survey year (ref: 2004)				
2006	0.40(−13.74,14.55)	−1.05(−15.42,13.31)	−3.31(−18.61,11.99)	−5.18(−20.7,10.34)
2009	12.98(−2.78,28.74)	12.64(−3.25,28.54)	10.15(−6.39,26.69)	9.97(−6.70,26.64)
2011	**−23.6(−9.14,-38.07)**	**−17.73(−1.77,-33.69)**	**−20.2(−5.28,-35.13)**	14.56(−1.95,31.06)
2006*Age	−0.10(−1.26,1.07)	−0.08(−1.25,1.1)	0.41(−0.84,1.66)	0.40(−0.86,1.66)
2009*Age	−1.04(−2.37,0.29)	−1.01(−2.35,0.34)	−0.60(−1.98,0.79)	−0.65(−2.05,0.76)
2011*Age	**−1.91(−3.15,-0.68)**	−1.20(−2.55,0.15)	**−1.31(−2.57,-0.05)**	**−0.96(−2.04,-0.73)**
Gender	**−6.38(−9.85,-2.9)**	**−5.86(−9.52,-2.2)**	**−6.24(−9.73,-2.75)**	**−5.74(−9.42,-2.06)**
Inverse Mill’s Ratio		3.99(−1.51,9.48)		**5.80(0.06,11.53)**
Community Urbanicity		**−0.15(−0.24,-0.05)**		**−0.07(−0.18,-0.03)**
Bicycle ownership			2.07(−1.24,5.37)	2.34(−1.12,5.79)
Vehicle ownership			0.71(−2.58,4.01)	0.06(−3.42,3.53)
No of Computers			**−4.80(−9.03,-0.57)**	**−4.74(−9.43,-0.05)**
No of Household members			0.71(−0.57,1.99)	0.41(−0.95,1.77)
Overweight/Obese			0.61(−3.91,5.14)	2.88(−1.97,7.72)
Household income			0.60(−1.68,2.89)	0.65(−1.79,3.08)
Number of observations	5697	5319	4532	4177
Number of individuals	3528	3162	3022	2676

Notes: Data from CHNS 2004–2011; bold values denote statistical significance at *p* < 0.05

In comparing the coefficients among models, we demonstrated that the number of computers per family was significantly higher and accounted for most of the period effects besides age and gender.

### Sedentary behaviour (SB)

**Fig. 3:**
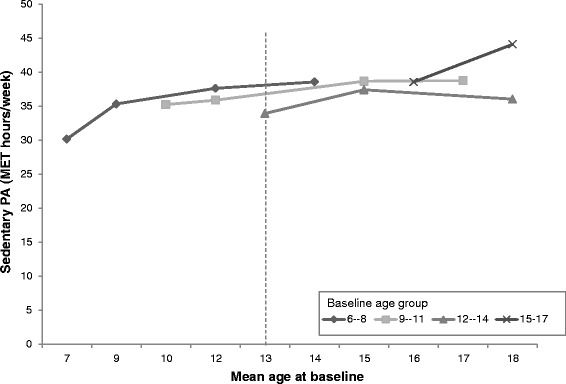
Age trends in mean Sedentary behaviour among boys in the CHNS by baseline age groups (MET-hrs/wk). Notes: Points represent Sedentary behaviour level of boys at the mean age for the age group in each survey year (2004, 2006, 2009, 2011). The vertical dotted line illustrates the difference in estimated PA level for a 13-year old boy in 2004 vs 13-year old boy in 2006 vs a 13-year old boy in 2009 (cohort effect)

SB levels of boys in the four baseline age groups are shown according to the mean age of the group by each survey year in Fig. 3. Each point represents a survey year and the lines span the 8-year period over which the PA data was collected. Younger boys had lower initial SB, and with age, PA levels increased across all baseline age groups. However, the relationship between age and mean SB is non-linear. Meanwhile, the vertical dotted line illustrates the difference in estimated PA level for 13-year old boys in 2004 (33 MET-hr./wk) compared to 13-year old boys in 2006 (36 MET-hr./wk), and in 2011 (37 MET-hr./wk). This is evidence of the cohort effect.

**Fig. 4:**
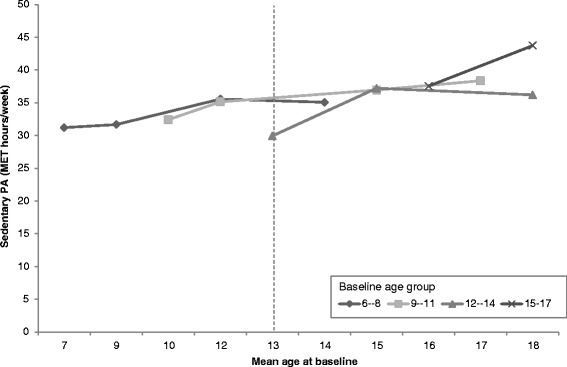
Age trends in mean Sedentary behaviour among girls in the CHNS by baseline age groups (MET-hrs/wk). Notes: Points represent Sedentary behaviour level of female children at the mean age for the age group in each survey year (2004, 2006, 2009, 2011). The vertical dotted line illustrates the difference in estimated PA level for a 13-year old girl in 2004 vs 13-year old girl in 2006 vs a 13-year old girl in 2009 (cohort effect)

The SB levels of girls in the four baseline age groups are also shown according to the mean age of the group by each survey year in Fig. 4. A similar trend was observed to the boys in that the level of SB increased by cohort year.

The non-linear age-related patterns of reductions in SB were identified by the longitudinal analysis. Higher age was positively associated with SB but gender was not associated with SB. The model results showed that time period was positively associated with PA in 2009, when controlling for age and gender (Table 3, model 1). The interaction between survey year and age (*p* < 0.05) was observed in 2006 (Table 3, model 1). However, the effect disappeared in model 4. In model 2, higher community urbanicity was found as a positive predictor of SB (Table 3, model 2). In model 3, we found that the number of computers owned and higher household incomes were positively associated with SB. In contrast, larger household size was a negative predictor of SB. After adjusting for potential confounding the effects of community urbanicity, number of computers per family, household size, and household income were identified as positive predictors of SB notwithstanding age (Table 3, model 4). Potential secular trends of SB was observed for both boys and girls (Additional file 1: Figure S1 and Additional file 2: Figure S2).

**Table 3:** Mixed effects estimates on sedentary behavior (SB) levels (MET-hrs/wk) among Chinese children, coefficient (95% CI)

SB	Model 1	Model 2	Model 3	Model 4
Age	**0.64(0.34,0.95)**	**0.53(0.22,0.84)**	**0.57(0.25,0.89)**	**0.49(0.17,0.81)**
Survey year (ref: 2004)				
2006	−1.99(−7.56,3.57)	−0.82(−6.45,4.8)	−1.14(−7,4.72)	−0.28(−6.22,5.66)
2009	**7.41(1.43,13.38)**	**6.8(0.82,12.79)**	**6.54(0.39,12.69)**	5.86(−0.33,12.04)
2011	2.97(−2.4,8.34)	0.86(−5.04,6.75)	2.61(−2.85,8.08)	1.24(−4.79,7.27)
2006*Age	**0.45(0.00,0.89)**	**0.45(0.00,0.9)**	0.37(−0.10,0.84)	0.39(−0.09,0.86)
2009*Age	−0.26(−0.75,0.23)	−0.19(−0.69,0.3)	−0.25(−0.75,0.26)	−0.15(−0.66,0.36)
2011*Age	0.30(−0.14,0.74)	0.23(−0.26,0.71)	0.16(−0.29,0.61)	0.12(−0.37,0.61)
Gender	−0.95(−2.12,0.22)	−0.89(−2.1,0.32)	−0.69(−1.86,0.49)	−0.54(−1.77,0.69)
Inverse Mill’s Ratio		**−3.64(−5.61,-1.68)**		**−3.21(−5.25,-1.17)**
Community Urbanicity		**0.11(0.08,0.14)**		**0.06(0.03,0.10)**
Bicycle ownership			0.50(−0.68,1.68)	0.37(−0.86,1.6)
Vehicle ownership			−0.87(−2.04,0.31)	0.06(−1.18,1.3)
No of Computers			**3.43(2.28,4.58)**	**2.41(1.12,3.71)**
No of Household members			**−1.12(−1.56,-0.67)**	**−0.89(−1.36,-0.42)**
Overweight/Obese			**1.32(0.00,2.64)**	1.5(−0.14,3.13)
Household income			**1.45(0.63,2.28)**	**0.93(0.04,1.81)**
Number of observations	5697	5319	4532	4177
Number of individuals	3528	3162	3022	2676

Notes: Data from CHNS 2004–2011; bold values denote statistical significance at *p* < 0.05

## Discussion

This study included Chinese children aged 6–17 years old between 2004 and 2011, and attempted to elucidate the epidemiological importance of age, period, and cohort changes in primary sources of both PA and SB, using a reliable, validated PA questionnaire [34–37].

Throughout this 8-year period, we found that PA levels decreased slightly but that SB moderately increased for both boys and girls. The proportion of SB from total PA was more pronounced in girls than boys and increased in both gender groups across time. Being older was associated with SB while being female was negatively associated with PA but not with SB. For PA, higher community urbanicity, more computers per family, and small period effects were found to be negative predictors of SB. We found that higher community urbanization, increased computer appliance ownership, and higher household income were positively associated with SB. Surprisingly, larger household size was found to be the only factor that was negatively associated with SB.

The CHNS was designed to provide representation of rural, urban, and suburban areas that vary substantially in geography, economic development, public resources, and health indicators. It is the only large-scale, longitudinal study of its kind in China [23]. It mainly focuses on household- and individual-level socio-demographic factors, diet, physical activity, health and behavioural changes within the context of community-level urbanicity, and social and economic change [22].

Accumulating evidence shows that higher levels of SB are associated with a higher risk of obesity, less healthy diet, and lower cardiopulmonary fitness in children and adolescents [38–40]. It is important to independently assess PA levels separately from SB. The PA levels of Chinese children have not changed significantly from 2004 to 2011. The government of China established a regulation to promote one hour of exercise to school students since 2007 [41]. This was done to prevent a dramatic decline in PA since most children and adolescents are school students.

Our study found that PA levels only slightly declined but that SB increased by 18.5% from 2004 to 2011. Several other studies corroborate that there is an increase in SB [1, 42, 43]. A previous study found that Chinese students are under extreme pressure to perform well in academic settings [44]. They spend more time on homework or other sedentary learning activities (e.g., drawing and playing chess). In another study, the cohorts experienced rapid environmental change at different ages with changes related to China’s dramatic economic, demographic, and social transformation. These changes included modernization of the Chinese food system, continued use and greater dissemination of modern technology in manufacturing, transportation and leisure, environmental and social systems, and changes to health system and educational systems [45, 46]. Moreover, technological advances in travel and leisure time have reduced the physical demands in many aspects. Those changes might result in an improved level of urbanisation, varying the way people live, while ultimately increasing SB levels across time.

The gross domestic product (GDP) of China increased 3.3-fold from 2004 to 2011 and the urbanization rate raised increased by nearly 10% in 8 years (from 41.8% in 2004 to 51.3% in 2011) [47]. Our data showed that the greater the level of community urbanization, the more likely this would be associated with higher levels of SB, and lower levels of PA. Increased community urbanization was associated with an increase in less laborious transportation, easier access to TV and computer appliances, and augmented academic pressure for Chinese children, which were all related to increased levels of SB and decreased PA, respectively.

The proportion of Chinese families who own colour televisions, have access to cable networks and computers has all also increased dramatically, as has screen time [48–50]. We initially included TV and computer ownership as a potential covariate into the models, however more than 90% of Chinese families owned a TV between 2004 and 2011. Thus, we did not believe it was not necessary to include TV ownership. Interestingly, we found that increased computer ownership was associated with the decline of PA in children, which conflicts with some other studies assessing the role of computer use [1, 21, 40]. On the contrary, increased level of computer ownership was associated with an increase of SB in Chinese children.

We found that being overweight or obese was marginally associated with higher SB. This result was consistent with some other studies [1, 3]. Weight status was related to other aforementioned factors described previously. Higher household income was associated with a lower level of SB, which was consistent with another study [51].

To our surprise, large household size was found to be the only factor that was negatively associated with SB in Chinese children. The reason for this might be due to family or siblings encouraging higher levels of physical activities [52]. Many Chinese families have only been able to have one child as a result of the ‘one-child’ policy since 1979 (which was only recently altered) [53]. Consequently, it is possible that single children have been overprotected by their parents in all aspects when compared to families with two children or more, and this may result in increased SB for only children. Besides, children with siblings may have someone to play with and influence each other [54]. We further analyzed the PA levels among single children and children with siblings. The results showed that PA levels were 46.2 ± 58.4 MET-hr./wk., 53.9 ± 76.8 MET-hr./wk., and 66.8 ± 92.9 MET-hr./wk. for a single child, children with one sibling, and children with more than one sibling, respectively. Moreover, the SB levels were 36.6 ± 21.9 MET-hr./wk., 32.9 ± 22.3 MET-hr./wk., and 28.0 ± 18.8 MET-hr./wk. for a single child, children with one sibling, and children with more than one sibling, respectively. From January 1, 2016, all Chinese couples are allowed to have two children. This marked the end of China’s ‘one-child’ policy, which had restricted the majority of Chinese families to have only one child for more than 35 years. Previously, we analyzed the PA level of adult females using CHNS data from 1991 to 2011 and we also found that bigger household size was positively associated with work and domestic PA [55]. The change in policy might positively influence children’s PA levels in a positive manner and might promote good health for both children and mothers.

Girls had lower PA levels than boys but SB levels were no different. However, the SB level of boys and girls was similar and the increasing trends were also similar for both genders. A previous study reported that SB was easier for girls to control but this was not the case for boys [56]. The study indicated that if girls and boys had the same SB levels, more attention should be focused on boys since overweight and obese male adults are more likely to suffer poor cardiovascular health compared with their female counterparts.

An important strength of this study is that this is the largest longitudinal analysis of physical activity to be conducted in a diverse sample of Chinese children. We separately assessed PA and SB changes among Chinese children while differentiating between effects of age, time period, and birth cohort. This allowed us to robustly determine how physical activity is influenced by biological, behavioral, economic, and environmental factors over time. To our knowledge, we believe this is the first study to assess these phenomena and reveal several important predictors for both PA and SB. The findings of our study may shed light on design of urbanization for policy-makers, while endorsing government-initiated health promotion and recommendation of physical activity for school age children.

Nonetheless, there are several limitations to this study. First, recall and social desirability biases exist in self-reported PA data, although it is not clear whether PA is overestimated due to social desirability bias in some developed countries [57]. Second, the collection methods of PA data in children and adolescents varied slightly by age groups, with assistance from caregivers for children <10 years. While different data collection methods were used to improve accuracy of PA, [58] comparisons with CHNS PA data in children based on parent-assisted self-reports and self-reports has not yet been conducted. Thirdly, we did not assess moderate to vigorous physical activity in our study, which may be more significant for public health issues. We separately analyzed physical activity as the physical activity and SB in our study. It is likely that moderate to vigorous physical activity MET hours contributed to most of the total PA MET hours.

Poor PA, especially physical activity, contributes to between 12 and 19% of the five major NCDs in China and the direct and indirect costs of physical inactivity in China has been estimated at 6.7 billion USD (in 2007 dollars) [59]. This cost is expected to grow as PA levels continue to decrease, given the potential age effect and secular trends observed in this study, as well as the much higher SB levels and lower PA levels of the younger cohorts.

## Conclusion

In summary, this study followed a large cohort of children over a significant period of their childhood. As China underwent dramatic economic transition, we observed potential age and secular trends in PA levels. Community urbanisation, computer ownership, and household size were related to PA levels. These findings are highly relevant for health policy and preventative health measures in China and other countries that are now facing similar public health challenges.

## Additional files

Additional file 1: Figure S1. Secular trends in SB level among boys in CHNS by baseline age groups. Notes: Bars represent difference from baseline (2004) Sedentary Activity, estimated from longitudinal models, stratified by baseline age groups. (PDF 285 kb)

Additional file 2: Figure S2. Secular trends in SB level among girls in CHNS by baseline age groups. Notes: Bars represent difference from baseline (2004) Sedentary Activity, estimated from longitudinal models, stratified by baseline age groups. (PDF 248 kb)

## Abbreviations

PA: Physical activity
CHNS: China Health and Nutrition Survey
MET: Metabolic equivalent of task
MET-hr./wk.: MET hours per week
DALYs: Disability-adjusted life-years
NCDs: Non-communicable diseases
APC: Age-period-cohort
