# Supplementary material for: Age, period and cohort effects and the predictors of physical activity and sedentary behaviour among Chinese children, from 2004 to 2011
Source: BMC Public Health. 2017 Apr 24;17:353. doi: 10.1186/s12889-017-4215-x (PMC5402654; doi:10.1186/s12889-017-4215-x)

Additional file 2: Figure S2. Secular trends in Sedentary Activity level among girls in CHNS by baseline age groups. Notes: Bars represent difference from baseline (2004) Sedentary Activity , estimated from longitudinal models, stratified by baseline age groups

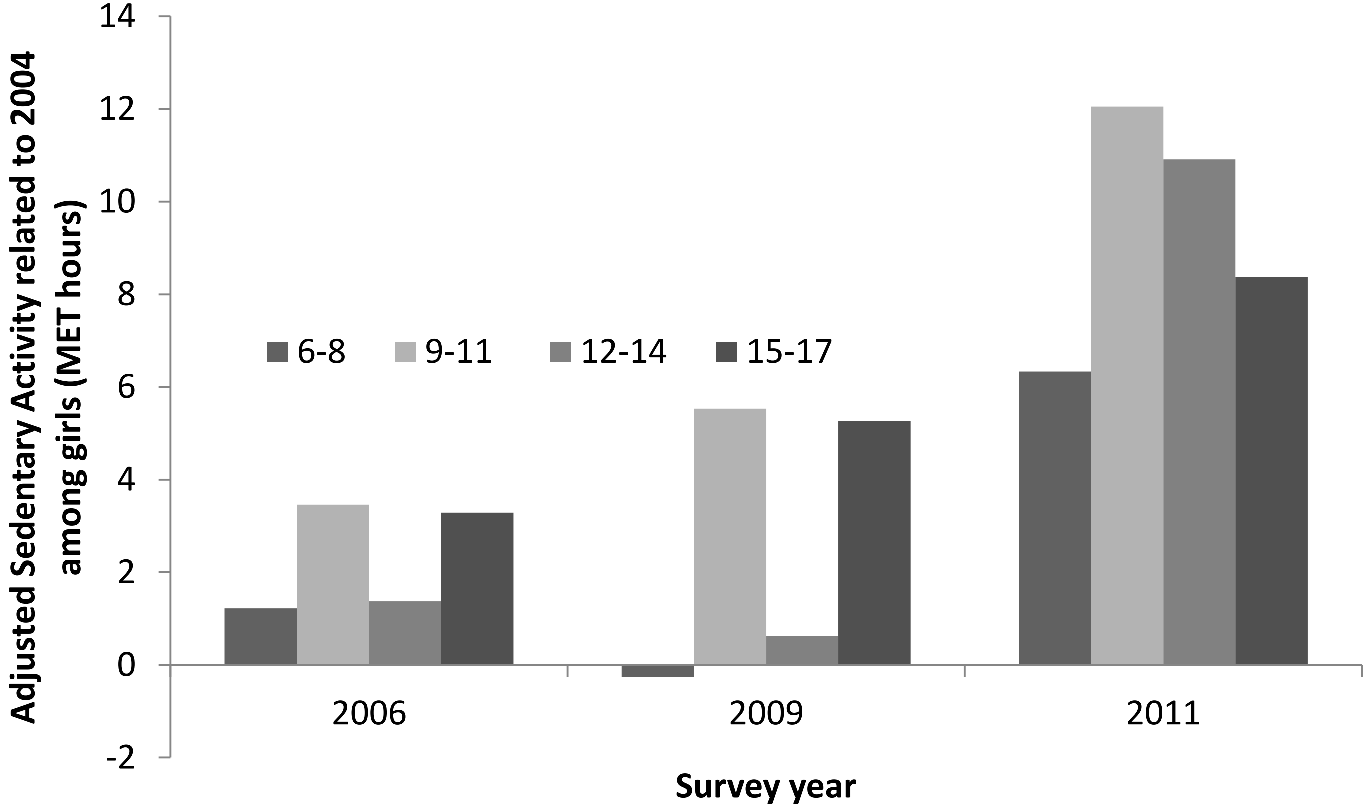

Supplement: Supplementary file 2 — Secular trends in SB level among girls in CHNS by baseline age groups. Notes: Bars represent difference from baseline (2004) Sedentary Activity, estimated from longitudinal models, stratified by baseline age groups. (PDF 248 kb) [file 12889_2017_4215_MOESM2_ESM.pdf]
